# Supplementary material for: Association between non-invasive biomarkers and quality of life in Primary Sclerosing Cholangitis
Source: PLoS One. 2025 Nov 12;20(11):e0335642. doi: 10.1371/journal.pone.0335642 (PMC12611166; doi:10.1371/journal.pone.0335642)
Supplement: S1 Table — (PDF) [file pone.0335642.s001.pdf]

S1 Table. Type of Data collected

Type	***Collection time point***	***Details***
Clinical data	Baseline and Year 1	Age, sex, PSC-duration, ursodeoxycholic acid (UDCA) use, inflammatory bowel disease (IBD) presence
Cholestasis marker	Baseline and Year 1	Upper limit normal of ALP (xULN ALP)
Prognostic models	Baseline and Year 1	Mayo risk score (MRS), Amsterdam-Oxford model (AOM)
Liver fibrosis markers	Baseline and Year 1	FibroScan liver stiffness (LS), enhanced liver fibrosis (ELF)
Qualitative MRI-MRCP reads	Baseline	Extrahepatic disease, dominant stricture, cirrhosis, portal hypertension, splenomegaly, hepatic dysmorphy, Anali score
Quantitative MRCP+ metrics	Baseline and Year 1	Intrahepatic and extrahepatic biliary tree metrics
LiverMultiScan metrics	Baseline and Year 1	Iron-corrected T1 (cT1)
Patient reported outcome measures (PROMs) instruments	Baseline and Year 1	RAND SF-36 and PSC-PRO
