# Supplementary material for: Association between non-invasive biomarkers and quality of life in Primary Sclerosing Cholangitis
Source: PLoS One. 2025 Nov 12;20(11):e0335642. doi: 10.1371/journal.pone.0335642 (PMC12611166; doi:10.1371/journal.pone.0335642)
Supplement: S1 Text — (PDF) [file pone.0335642.s002.pdf]

S1 Text. Bootstrap approach to assess robustness of findings

We used a bootstrap approach to test the robustness of the findings. First, we bootstrapped the sample 1000 times, re-estimating the regression for each sample, and recording p-value for each covariate. We then estimated the number of times that the p-value for the covariate of interest was less than 0.05. We judged the results to be consistent if a coefficient was significant for more than 50% of the bootstrap samples.
