# Supplementary material for: Association between non-invasive biomarkers and quality of life in Primary Sclerosing Cholangitis
Source: PLoS One. 2025 Nov 12;20(11):e0335642. doi: 10.1371/journal.pone.0335642 (PMC12611166; doi:10.1371/journal.pone.0335642)
Supplement: S2 Text — (PDF) [file pone.0335642.s003.pdf]

S2 Text. Cook's distance to identify potential outliers

For the predictors that passed the bootstrap criteria, we used the Cook's distance to identify potential outliers and adjust our models. This approach tests the impact of removing one observation from the original sample, in case there are only few data points are driving the results in the regression. We removed the observations with top 5 largest Cook's distance values then re-estimate the models. The final predictors had to remain significant after removing outliers.
