# Supplementary material for: Association between non-invasive biomarkers and quality of life in Primary Sclerosing Cholangitis
Source: PLoS One. 2025 Nov 12;20(11):e0335642. doi: 10.1371/journal.pone.0335642 (PMC12611166; doi:10.1371/journal.pone.0335642)
Supplement: S3 Text — (PDF) [file pone.0335642.s004.pdf]

S3 Text. Multiple imputation for sensitivity analysis

For the sensitivity analysis, multiple imputation (MI) was used to impute the missing PROMs at visit 1 and visit 2, used visit 1 biomarkers and other variables in imputation model (see table S2 below). Then we did the cross-sectional analysis with the imputed data set. We filled in the missing data by using the predictive mean matching with a 10 closest observation (knn=10).
