# Supplementary material for: Association between non-invasive biomarkers and quality of life in Primary Sclerosing Cholangitis
Source: PLoS One. 2025 Nov 12;20(11):e0335642. doi: 10.1371/journal.pone.0335642 (PMC12611166; doi:10.1371/journal.pone.0335642)
Supplement: S2 Table — (PDF) [file pone.0335642.s005.pdf]

S2 Table. Imputation Model

Variables to be imputed	Imputation model
**Visit 1 and Visit 2:**	**Visit 1:**
PSC PRO symptoms,	Ethnicity
PSC PRO total impact of symptom,	Age
SF36 Physical component summary,	Sex
SF36 Mental component summary,	Duration
SF 6D quality of life	IBD presence
	xULNALP
	MRS
	AOM
	LS
	ELF
	cT1 n
	Anali
	Extrahepatic disease
	Dominant stricture
	cirrhosis
