# Supplementary figures and images for: Association between non-invasive biomarkers and quality of life in Primary Sclerosing Cholangitis
Source: PLoS One. 2025 Nov 12;20(11):e0335642. doi: 10.1371/journal.pone.0335642 (PMC12611166; doi:10.1371/journal.pone.0335642)

S1 Fig. Study Flowchart

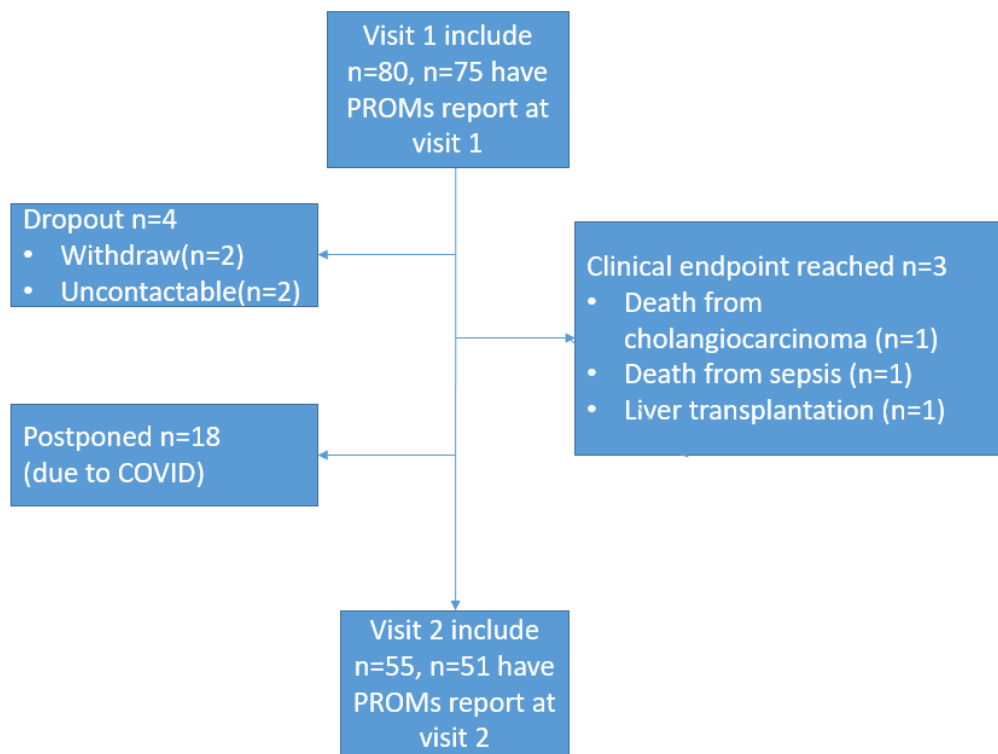

Supplement: S1 Fig — (PDF) [file pone.0335642.s006.pdf]
