# Supplementary material for: Association between non-invasive biomarkers and quality of life in Primary Sclerosing Cholangitis
Source: PLoS One. 2025 Nov 12;20(11):e0335642. doi: 10.1371/journal.pone.0335642 (PMC12611166; doi:10.1371/journal.pone.0335642)
Supplement: S3 Table — (PDF) [file pone.0335642.s007.pdf]

S3 Table. Descriptive statistics and comparison between different sample sizes

	Recruited sample	Recruited sample with PROMs at baseline	Recruited sample with PROMs at baseline and year 1 (analysis sample)	Difference between Recruited sample and analysis sample, p-value †
N	80	75	51	(80 vs 51)
White, n(%)	72 (90.0%)	68 (90.7%)	47 (92.2%)	0.677
Male, n(%)	54 (67.5%)	51 (68.0%)	34 (66.7%)	0.921
IBD presence, n(%)	61 (76.2%)	57 (76.0%)	34 (66.7%)	0.231
Age, mean(SD)	43.3 (16.7)	42.5 (16.2)	44.9 (15.8)	0.595
PSC duration, mean(SD)	8.5 (5.9)	8.3 (5.7)	9.0 (6.3)	0.636
LSM, mean(SD)	10.3 (8.5)	10.5 (8.7)	9.5 (6.9)	0.566
ELFS, mean(SD)	9.6 (1.2)	9.6 (1.1)	9.5 (1.0)	0.588
xULN ALP, mean(SD)	1.5 (1.3)	1.6 (1.4)	1.3 (0.9)	0.317
AOM , mean(SD)	1.7 (0.7)	1.7 (0.7)	1.7 (0.6)	0.947
MRS, mean(SD)	0.1 (0.8)	0.1 (0.8)	0.1 (0.6)	0.652

† For proportion report: white; male; IBD presence, test of proportion was used to test the difference in different samples' population proportions. For continuous variables' mean (SD) report: Age, LSM, ELFS, ALP, AOM, MRS, unpaired t-test was used to test the difference.
