# Supplementary material for: Association between non-invasive biomarkers and quality of life in Primary Sclerosing Cholangitis
Source: PLoS One. 2025 Nov 12;20(11):e0335642. doi: 10.1371/journal.pone.0335642 (PMC12611166; doi:10.1371/journal.pone.0335642)
Supplement: S4 Table — (PDF) [file pone.0335642.s008.pdf]

S4 Table. PROMs comparison between different sample sizes

Variable	Baseline	Baseline	Mean Difference between baseline	Year 1	Mean Difference between baseline and year 1 (p-value)
	Recruited sample with PROMs at baseline	Recruited sample with PROMs at baseline and year 1 (analysis sample)	recruited sample and analysis sample	Recruited sample with PROMs at baseline and year 1 (analysis sample)	Recruited sample with PROMs at baseline and year 1 (analysis sample)
N	75	51	75 vs. 51	51	51
SF6D Quality of life	0.81(0.15) †	0.81 (0.14)	0.01(0.79)	0.80 (0.16)	0.01(0.73)
SF36: Physical component summary	49.66 (8.29)	50.02 (8.06)	-0.14(0.93)	48.79 (9.83)	1.23(0.48)
SF36: Mental component summary	49.11 (10.55)	49.03 (11.30)	0.35(0.86)	48.39 (11.98)	0.64(0.78)
PSC-PRO: PSC Symptoms	1.97 (10.55)	2.85 (12.69)	-1.05(0.62)	2.74 (7.81)	0.11(0.96)
PSC-PRO: Total Impact of Symptoms	8.96 (3.38)	9.24 (3.46)	-0.39(0.54)	9.65 (3.91)	-0.41(0.57)
