# Supplementary material for: Association between non-invasive biomarkers and quality of life in Primary Sclerosing Cholangitis
Source: PLoS One. 2025 Nov 12;20(11):e0335642. doi: 10.1371/journal.pone.0335642 (PMC12611166; doi:10.1371/journal.pone.0335642)
Supplement: S5 Table — (PDF) [file pone.0335642.s009.pdf]

S5 Table. The within difference of patients' PROMs

Variable		Mean	Std. dev.	Min	Max	Observations
SF6D QoL	overall	0.807	0.149	0.491	1.000	N = 96
	between		0.143	0.560	0.983	n = 48
	within		0.044	0.673	0.941	T = 2
SF36 PCS	overall	49.392	9.062	25.548	60.547	N = 100
	between		8.428	30.316	60.375	n = 50
	within		3.438	40.116	58.668	T = 2
SF36 MCS	overall	48.649	11.617	11.634	64.840	N = 100
	between		11.213	22.868	64.097	n = 50
	within		3.240	37.415	59.884	T = 2
PSC-PRO Symptoms	overall	2.969	10.783	0.000	66.000	N = 96
	between		8.397	0.000	41.000	n = 48
	within		6.820	-30.031	35.969	T = 2
PSC-PRO Total Impact of Symptoms	overall	9.523	3.715	6.000	21.500	N = 96
	between		3.502	6.000	19.500	n = 48
	within		1.291	4.148	14.898	T = 2
