# Supplementary material for: Association between non-invasive biomarkers and quality of life in Primary Sclerosing Cholangitis
Source: PLoS One. 2025 Nov 12;20(11):e0335642. doi: 10.1371/journal.pone.0335642 (PMC12611166; doi:10.1371/journal.pone.0335642)
Supplement: S6 Table — (PDF) [file pone.0335642.s010.pdf]

S6 Table. Baseline PROM scores by risk group

Baseline year PROMs by different risk groups					
Baseline risk group	SF6D QoL	PCS	MCS	PSC-PRO symptoms	PSC-PRO total impact of symptoms
**Extrahepatic disease**					
Coefficient	-0.022	-2.873	0.026	3.610	0.599
p-value	0.524	0.146	0.992	0.141	0.448
CI	[-0.090 0.046]	[-6.770 1.024]	[-5.017 5.068]	[-1.228 8.447]	[-0.965 2.164]
**Dominant stricture**					
Coefficient	-0.048	-3.625	-0.252	**5.800**	0.340
p-value	0.193	0.081	0.925	**0.024**	0.684
CI	[-0.120 0.025]	[-7.714 0.464]	[-5.577 5.072]	**[0.794 10.806]**	[-1.317 1.997]
**ULNALP 1.5 risk**					
Coefficient	-0.043	-3.648	-1.575	0.747	1.150
p-value	0.234	0.077	0.553	0.773	0.162
CI	[-0.114 0.028]	[-7.696 0.399]	[-6.837 3.687]	[-4.386 5.881]	[-0.471 2.771]
**ULNALP 2.2 risk**					
Coefficient	-0.078	-4.870	-2.190	3.382	1.514
p-value	0.090	0.060	0.511	0.296	0.143
CI	[-0.168 0.013]	[-9.944 0.204]	[-8.802 4.422]	[-3.027 9.791]	[-0.521 3.549]
**MRS > 0**					
Coefficient	-0.012	-0.950	-0.914	2.442	-0.144
p-value	0.732	0.634	0.720	0.323	0.856
CI	[-0.080 0.057]	[-4.909 3.009]	[-5.966 4.139]	[-2.450 7.333]	[-1.718 1.431]
**AOM > 2**					
Coefficient	-0.059	-4.122	-3.142	5.181	1.385
p-value	0.111	0.052	0.249	0.049	0.102
CI	[-0.132 0.014]	[-8.281 0.037]	[-8.537 2.252]	[0.019 10.342]	[-0.280 3.050]
**LS_9_6**					
Coefficient	**-0.081**	**-4.996**	-5.043	1.042	1.275
p-value	**0.027**	**0.018**	0.063	0.696	0.132
CI	**[-0.153 -0.010]**	**[-9.103 - 0.890]**	[-10.358 0.273]	[-4.254 6.337]	[-0.394 2.945]
**ELF_9_8**					
Coefficient	-0.068	-3.789	-4.307	1.042	1.228
p-value	0.066	0.075	0.113	0.696	0.147
CI	[-0.141 0.005]	[-7.966 0.387]	[-9.657 1.044]	[-4.254 6.337]	[-0.443 2.900]
**RSIBD**					
Coefficient	**-0.081**	-3.009	-2.424	3.688	1.089
p-value	**0.016**	0.126	0.336	0.131	0.164

Baseline year PROMs by different risk groups					
Baseline risk group	SF6D QoL	PCS	MCS	PSC-PRO symptoms	PSC-PRO total impact of symptoms
CI	**[-0.147 -0.016]**	[-6.886 0.868]	[-7.417 2.569]	[-1.128 8.505]	[-0.455 2.634]
**IBD presence**					
Coefficient	0.019	-0.148	-0.646	-2.228	-1.198
p-value	0.636	0.949	0.827	0.438	0.191
CI	[-0.060 0.098]	[-4.756 4.461]	[-6.522 5.230]	[-7.928 3.472]	[-3.007 0.612]
**Anali**					
Coefficient	-0.008	-0.864	-1.425	0.736	0.588
p-value	0.823	0.676	0.588	0.774	0.473
CI	[-0.079 0.063]	[-4.959 3.232]	[-6.644 3.794]	[-4.353 5.826]	[-1.035 2.211]
**Cirrhosis**					
Coefficient	-0.003	-1.280	-1.840	1.624	0.441
p-value	0.928	0.561	0.513	0.553	0.614
CI	[-0.079 0.072]	[-5.654 3.093]	[-7.414 3.735]	[-3.807 7.055]	[-1.297 2.180]
**cT1 Whole Median**					
Coefficient	**-0.161**	**-10.595**	**-10.726**	0.660	0.399
p-value	**0.004**	**0.001**	**0.012**	0.876	0.768
CI	**[-0.271 -0.052]**	**[-16.894 -4.296]**	**[-18.985 -2.467]**	[-7.742 9.061]	[-2.288 3.085]
Number of observations	73	75	75	75	75
