# Supplementary material for: Association between non-invasive biomarkers and quality of life in Primary Sclerosing Cholangitis
Source: PLoS One. 2025 Nov 12;20(11):e0335642. doi: 10.1371/journal.pone.0335642 (PMC12611166; doi:10.1371/journal.pone.0335642)
Supplement: S7 Table — (PDF) [file pone.0335642.s011.pdf]

S7 Table. PROM scores at year 1 by baseline PSC disease severity group

	PROMs at year 1 visit				
Baseline disease severity	SF6D QoL	SF36 PCS	SF36 MCS	PSC-PRO symptoms	PSC-PRO total impact of symptom
**Extrahepatic disease**					
Coefficient	**-0.085**	**-5.750**	-2.929	**5.269**	1.741
p-value	**0.053**	**0.038**	0.395	**0.016**	0.117
CI	**[-0.171 0.001]**	**[-11.163 - 0.337]**	[-9.794 3.936]	**[1.046 9.493]**	[-0.450 3.932]
**Dominant stricture**					
Coefficient	**-0.101**	**-8.394**	-4.096	**4.583**	**2.850**
p-value	**0.029**	**0.003**	0.258	**0.048**	**0.013**
CI	**[-0.191 - 0.011]**	**[-13.848 - 2.939]**	[-11.294 3.102]	**[0.036 9.130]**	**[0.628 5.072]**
**ULNALP 1.5 risk**					
Coefficient	-0.027	-2.271	-1.562	4.821	0.796
p-value	0.594	0.480	0.692	0.055	0.533
CI	[-0.129 0.075]	[-8.688 4.147]	[-9.427 6.304]	[-0.100 9.742]	[-1.755 3.346]
**ULNALP 2.2 risk**					
Coefficient	0.008	0.508	2.623	**6.735**	0.258
p-value	0.907	0.907	0.621	**0.046**	0.882
CI	[-0.130 0.146]	[-8.199 9.215]	[-7.984 13.231]	**[0.112 13.358]**	[-3.198 3.713]
**MRS > 0**					
Coefficient	-0.015	-2.479	-0.191	1.080	1.197
p-value	0.745	0.379	0.956	0.630	0.284
CI	[-0.104 0.075]	[-8.092 3.135]	[-7.103 6.721]	[-3.397 5.557]	[-1.023 3.416]
**AOM > 2**					
Coefficient	-0.022	-3.669	0.058	3.895	1.574
p-value	0.650	0.231	0.988	0.107	0.195
CI	[-0.120 0.075]	[-9.751 2.414]	[-7.483 7.600]	[-0.869 8.660]	[-0.834 3.982]
**LS_9_6**					
Coefficient	-0.092	**-6.536**	**-7.800**	3.895	**2.915**
p-value	0.054	**0.030**	**0.034**	0.107	**0.014**
CI	[-0.186 0.002]	**[-12.412 - 0.659]**	**[-14.994 - 0.607]**	[-0.869 8.660]	**[0.614 5.216]**
**ELF_9_8**					
Coefficient	-0.060	-5.004	-5.037	3.895	**3.018**
p-value	0.219	0.100	0.177	0.107	**0.011**

	PROMs at year 1 visit				
Baseline disease severity	SF6D QoL	SF36 PCS	SF36 MCS	PSC-PRO symptoms	PSC-PRO total impact of symptom
CI	[-0.156 0.037]	[-11.006 0.998]	[-12.435 2.362]	[-0.869 8.660]	**[0.729 5.308]**
**RSIBD**					
Coefficient	-0.068	-4.908	-0.677	3.640	1.223
p-value	0.126	0.078	0.845	0.100	0.273
CI	[-0.155 0.020]	[-10.385 0.570]	[-7.586 6.232]	[-0.722 8.002]	[-0.995 3.442]
**IBD presence**					
Coefficient	0.002	1.553	1.491	4.152	-0.763
p-value	0.973	0.603	0.682	0.075	0.519
CI	[-0.093 0.096]	[-4.404 7.510]	[-5.792 8.774]	[-0.430 8.733]	[-3.124 1.598]
**Anali**					
Coefficient	-0.034	-4.112	-2.519	1.374	1.825
p-value	0.475	0.164	0.489	0.561	0.119
CI	[-0.128 0.060]	[-9.965 1.742]	[-9.778 4.740]	[-3.346 6.095]	[-0.486 4.137]
**Cirrhosis**					
Coefficient	-0.017	-3.217	-1.074	-1.088	0.892
p-value	0.726	0.286	0.772	0.651	0.457
CI	[-0.113 0.079]	[-9.211 2.777]	[-8.476 6.328]	[-5.889 3.712]	[-1.502 3.287]
**cT1**					
Coefficient	**-0.190**	**-15.489**	-7.209	**11.659**	**3.682**
p-value	**0.004**	**<0.001**	0.171	**<0.001**	**0.029**
CI	**[-0.316 -0.063]**	**[-22.948 -8.031]**	[-17.636 3.218]	**[5.639 17.679]**	**[0.395 6.970]**
Number of observations	50	50	50	50	50
