# Supplementary material for: Association between non-invasive biomarkers and quality of life in Primary Sclerosing Cholangitis
Source: PLoS One. 2025 Nov 12;20(11):e0335642. doi: 10.1371/journal.pone.0335642 (PMC12611166; doi:10.1371/journal.pone.0335642)
Supplement: S8 Table — (PDF) [file pone.0335642.s012.pdf]

S8 Table. Change in PROM scores by baseline PSC severity

	Change of PROMs between baseline and year 1				
	SF 6D	SF36 PCS	SF36 MCS	PSC-PRO symptoms	PSC-PRO total impact of Symptoms
**Extrahepatic disease**					
Coefficient	-0.037	-0.344	-1.053	-0.440	0.228
p-value	0.143	0.861	0.577	0.913	0.760
CI	[-0.087 0.013]	[-4.267 3.578]	[-4.827 2.721]	[-8.499 7.619]	[-1.265 1.721]
**Dominant stricture**					
Coefficient	-0.009	-2.041	-1.142	-4.017	0.783
p-value	0.730	0.321	0.567	0.337	0.313
CI	[-0.064 0.045]	[-6.137 2.054]	[-5.122 2.837]	[-12.351 4.317]	[-0.761 2.327]
**ULNALP 1.5 risk**					
Coefficient	0.038	1.909	-0.052	2.213	-0.327
p-value	0.188	0.391	0.981	0.624	0.697
CI	[-0.020 0.097]	[-2.525 6.344]	[-4.365 4.260]	[-6.823 11.250]	[-2.004 1.351]
**ULNALP 2.2 risk**					
Coefficient	**0.081**	5.552	2.405	-1.643	-0.778
p-value	**0.048**	0.061	0.407	0.787	0.489
CI	**[0.001 0.161]**	[-0.262 11.365]	[-3.374 8.184]	[-13.808 10.522]	[-3.024 1.468]
**MRS > 0**					
Coefficient	-0.016	-2.632	-0.973	-3.400	1.100
p-value	0.541	0.175	0.606	0.397	0.136
CI	[-0.067 0.035]	[-6.478 1.213]	[-4.746 2.799]	[-11.396 4.596]	[-0.358 2.559]
**AOM > 2**					
Coefficient	0.005	-0.445	-0.673	-4.315	0.619
p-value	0.856	0.835	0.744	0.327	0.449
CI	[-0.051 0.061]	[-4.720 3.831]	[-4.796 3.451]	[-13.080 4.450]	[-1.013 2.251]
**LS_9_6**					
Coefficient	0.009	-0.629	-2.754	2.340	1.459
p-value	0.748	0.768	0.178	0.596	0.070
CI	[-0.047 0.065]	[-4.903 3.645]	[-6.804 1.295]	[-6.491 11.171]	[-0.125 3.044]
**ELF_9_8**					
Coefficient	-0.021	-1.507	-2.981	2.340	**2.098**
p-value	0.450	0.480	0.144	0.596	**0.008**
CI	[-0.077 0.035]	[-5.763 2.748]	[-7.017 1.055]	[-6.491 11.171]	**[0.578 3.618]**
**RSIBD**					
Coefficient	0.017	-0.806	2.504	-2.208	-0.389

Change of PROMs between baseline and year 1					
	SF 6D	SF36 PCS	SF36 MCS	PSC-PRO symptoms	PSC-PRO total impact of Symptoms
p-value	0.503	0.681	0.181	0.582	0.602
CI	[-0.034 0.068]	[-4.720 3.108]	[-1.209 6.217]	[-10.234 5.818]	[-1.878 1.100]
**IBD presence**					
Coefficient	0.000	3.071	4.091	5.844	0.076
p-value	0.989	0.133	0.036	0.166	0.924
CI	[-0.054 0.053]	[-0.970 7.112]	[0.278 7.904]	[-2.520 14.207]	[-1.508 1.659]
**Anali**					
Coefficient	-0.019	-1.963	0.149	-0.055	1.014
p-value	0.488	0.340	0.941	0.990	0.203
CI	[-0.073 0.035]	[-6.062 2.136]	[-3.844 4.142]	[-8.741 8.632]	[-0.568 2.597]
**Cirrhosis**					
Coefficient	-0.017	-1.942	-0.516	-3.105	0.560
p-value	0.531	0.353	0.799	0.482	0.494
CI	[-0.072 0.038]	[-6.106 2.223]	[-4.568 3.536]	[-11.915 5.705]	[-1.074 2.194]
**cT1**					
Coefficient	**-0.072**	**-6.762**	-1.182	**13.651**	**3.854**
p-value	**0.059**	**0.021**	0.684	**0.034**	**0.001**
CI	**[-0.146 0.003]**	**[-12.467 -1.058]**	[-6.993 4.629]	**[1.109 26.194]**	**[1.694 6.014]**
Number of observations	48	50	50	48	48
