# Supplementary material for: Association between non-invasive biomarkers and quality of life in Primary Sclerosing Cholangitis
Source: PLoS One. 2025 Nov 12;20(11):e0335642. doi: 10.1371/journal.pone.0335642 (PMC12611166; doi:10.1371/journal.pone.0335642)
Supplement: S4 Text — (PDF) [file pone.0335642.s013.pdf]

S4 Text. Robustness check results

The univariate analysis bootstrap shows the 62.5% of chance that LS is significant correlation with SF-36 quality of life; 75.3% of chance LS is significant correlation with SF-36 physical component summary; 58.7% of chance LS is significant with SF-36 Mental component summary. The multivariate analysis bootstrap shows there are 80.7% chance LS random effects is significantly correlated with SF-36 physical component summary, and still significant after remove 5 outliners.

Extrahepatic DilatSevRelSum is negatively correlated with SF-36 Mental component summary (β=-0.031, p =0.016).

The MRI metric cT1 whole IQR has significant positive relationship with health outcome: SF36 PCS (β=0.134, p =0.004) and PSC-PRO PSC symptoms (β=-0.299, p = 0.014).

Extrahepatic AbnormalLengthSum has 31% chance of being significantly correlated with SF-36 Physical component summary; 52% chance that cT1 Whole IQR is significantly correlated with PSC-PRO Physical function. For the estimator has last than 50% chance of being significant, they are not significant if we remove 5 patients out of study sample after doing cook's distance analysis.

The multivariate analysis bootstrap shows there are 80.7% chance LS random effects is significantly correlated with SF-36 physical component summary, 73.1% chance cT1 whole IQR is significant. In SF-36 Mental component summary model, also the chance of been significant is below 50%, fixed effects estimator of Extrahepatic DilatSevRelSum is still significant after remove 5 outliner patients (β=0.028, p =0.034). Bootstrap results for SF-6D shows that LS fixed effects estimator has 63.7% to be significant, remain significant after remove outliners (β=-0.002, p-value<0.001). The bootstrap for estimator cT1 whole IQR shows 56.5% chance of being significant correlated with PSC-PRO PSC symptom and will not be significant after remove the outliners (β=-0.027, p-value=0.660).
