# Supplementary material for: Association between non-invasive biomarkers and quality of life in Primary Sclerosing Cholangitis
Source: PLoS One. 2025 Nov 12;20(11):e0335642. doi: 10.1371/journal.pone.0335642 (PMC12611166; doi:10.1371/journal.pone.0335642)
Supplement: S9 Table — (PDF) [file pone.0335642.s014.pdf]

S9 Table. Univariate analysis for other PROMs' domains

	LS	**EH_StricSevRelSum**	cT1 IQR
**SF6D QoL**	-0.002 ***ϯ		
**SF36 Physical component summary**	-0.246***		
**SF36 mental component summary**	-0.171***		
SF-36 physical functioning			
SF36 Role Physical	-0.969**		0.700 **
SF36 bodily pain			
SF36 General Health			
SF36 Vitality			
SF36 Social functioning		-1.161**	
SF36 role emotional			
SF36 Mental Health			
**PSC-PRO Symptoms**			
**PSC-PRO total impact of symptoms**			
PSC-PRO Physical Function			
PSC-PRO Activities of Daily Living			
PSC-PRO work productivity			
PSC-PRO Role Function			
PSC-PRO Emotional Impact			
PSC-PRO social leisure impact			
PSC-PRO quality of life			

ϯp<0.05 "*" p< 0.01 "**" p<0.001 "***"
