# Supplementary material for: Association between non-invasive biomarkers and quality of life in Primary Sclerosing Cholangitis
Source: PLoS One. 2025 Nov 12;20(11):e0335642. doi: 10.1371/journal.pone.0335642 (PMC12611166; doi:10.1371/journal.pone.0335642)
Supplement: S10 Table — (PDF) [file pone.0335642.s015.pdf]

S10 Table. Sensitivity analysis (Multiple imputation)

a).Cross-sectional data analysis (n=80)

Baseline year PROMs by different risk groups (Multiple imputation)

	SF6D QoL		SF36 physical component summary		SF36 mental component summary	
	Available data	MI	Available data	MI	Available data	MI
**Extrahepatic disease**						
Coefficient	-0.022	-0.019	-2.873	-2.782	0.026	-0.029
p-value	0.524	0.564	0.146	0.152	0.992	0.991
CI	[-0.090 0.046]	[-0.086 0.047]	[-6.770 1.024]	[-6.609 1.044]	[-5.017 5.068]	[-4.964 4.907]
**Dominant stricture**						
Coefficient	-0.048	-0.048	-3.625	-3.697	-0.252	-0.535
p-value	0.193	0.176	0.081	0.073	0.925	0.840
CI	[-0.120 0.025]	[-0.119 0.022]	[-7.714 0.464]	[-7.746 0.351]	[-5.577 5.072]	[-5.787 4.717]
**ULNALP 1.5 risk**						
Coefficient	-0.043	-0.052	-3.648	-3.667	-1.575	-1.753
p-value	0.234	0.139	0.077	0.072	0.553	0.501
CI	[-0.114 0.028]	[-0.120 0.017]	[-7.696 0.399]	[-7.665 0.330]	[-6.837 3.687]	[-6.923 3.416]
**ULNALP 2.2 risk**						
Coefficient	-0.078	**-0.088**	-4.870	-4.957	-2.190	-2.400
p-value	0.090	**0.048**	0.060	0.054	0.511	0.468
CI	[-0.168 0.013]	**[-0.176 - 0.001]**	[-9.944 0.204]	[-10.004 0.091]	[-8.802 4.422]	[-8.946 4.147]
**MRS > 0**						
Coefficient	-0.012	-0.007	-0.950	-0.667	-0.914	-0.557
p-value	0.732	0.844	0.634	0.734	0.720	0.824
CI	[-0.080 0.057]	[-0.073 0.060]	[-4.909 3.009]	[-4.568 3.234]	[-5.966 4.139]	[-5.515 4.402]
**AOM > 2**						
Coefficient	-0.059	-0.060	-4.122	-3.524	-3.142	-2.659
p-value	0.111	0.097	0.052	0.091	0.249	0.319
CI	[-0.132 0.014]	[-0.130 0.011]	[-8.281 0.037]	[-7.627 0.579]	[-8.537 2.252]	[-7.936 2.618]
**LS_9_6**						
Coefficient	**-0.081**	**-0.080**	**-4.996**	**-4.354**	-5.043	-4.463
p-value	**0.027**	**0.025**	**0.018**	**0.036**	0.063	0.092
CI	**[-0.153 - 0.010]**	**[-0.150 - 0.010]**	**[-9.103 - 0.890]**	**[-8.416 - 0.291]**	[-10.358 0.273]	[-9.678 0.752]
**ELF_9_8**						
Coefficient	-0.068	-0.065	-3.789	-2.876	-4.307	-3.670

	SF6D QoL		SF36 physical component summary		SF36 mental component summary	
	Available data	MI	Available data	MI	Available data	MI
p-value	0.066	0.067	0.075	0.165	0.113	0.166
CI	[-0.141 0.005]	[-0.135 0.005]	[-7.966 0.387]	[-6.963 1.212]	[-9.657 1.044]	[-8.897 1.556]
**RSIBD**						
Coefficient	**-0.081**	**-0.076**	-3.009	-2.730	-2.424	-2.301
p-value	**0.016**	**0.020**	0.126	0.157	0.336	0.350
CI	**[-0.147 - 0.016]**	**[-0.140 - 0.013]**	[-6.886 0.868]	[-6.540 1.079]	[-7.417 2.569]	[-7.178 2.576]
**IBD presence**						
Coefficient	0.019	0.013	-0.148	-0.634	-0.646	-1.054
p-value	0.636	0.738	0.949	0.779	0.827	0.714
CI	[-0.060 0.098]	[-0.064 0.089]	[-4.756 4.461]	[-5.123 3.855]	[-6.522 5.230]	[-6.756 4.648]
**Anali**						
Coefficient	-0.008	0.011	-0.864	-0.074	-1.425	-0.677
p-value	0.823	0.755	0.676	0.970	0.588	0.791
CI	[-0.079 0.063]	[-0.057 0.079]	[-4.959 3.232]	[-4.054 3.905]	[-6.644 3.794]	[-5.753 4.399]
**Cirrhosis**						
Coefficient	-0.003	0.008	-1.280	-0.582	-1.840	-0.906
p-value	0.928	0.815	0.561	0.785	0.513	0.738
CI	[-0.079 0.072]	[-0.064 0.081]	[-5.654 3.093]	[-4.829 3.664]	[-7.414 3.735]	[-6.293 4.480]
**cT1 Whole Median**						
Coefficient	**-0.161**	**-0.124**	**-10.595**	**-8.243**	**-10.726**	-7.809
p-value	**0.004**	**0.019**	**0.001**	**0.008**	**0.012**	0.051
CI	**[-0.271 - 0.052]**	**[-0.226 - 0.021]**	**[-16.894 - 4.296]**	**[-14.247 - 2.239]**	**[-18.985 - 2.467]**	[-15.640 0.022]
Number of observations	73	80	75	80	75	80

	PSC-PRO symptoms		PSC-PRO Total impact of symptoms	
	Available data	MI	Available data	MI
**Extrahepatic disease**				
Coefficient	3.610	3.498	0.599	0.622
p-value	0.141	0.130	0.448	0.416
CI	[-1.228 8.447]	[-1.048 8.043]	[-0.965 2.164]	[-0.894 2.137]
**Dominant stricture**				
Coefficient	**5.800**	**5.779**	0.340	0.408
p-value	**0.024**	**0.019**	0.684	0.617
CI	**[0.794 10.806]**	**[0.992 10.566]**	[-1.317 1.997]	[-1.210 2.027]

	PSC-PRO symptoms		PSC-PRO Total impact of symptoms	
	Available data	MI	Available data	MI
**ULNALP 1.5 risk**				
Coefficient	0.747	0.770	1.150	1.172
p-value	0.773	0.753	0.162	0.144
CI	[-4.386 5.881]	[-4.092 5.632]	[-0.471 2.771]	[-0.408 2.752]
**ULNALP 2.2 risk**				
Coefficient	3.382	3.465	1.514	1.559
p-value	0.296	0.268	0.143	0.124
CI	[-3.027 9.791]	[-2.714 9.645]	[-0.521 3.549]	[-0.439 3.557]
**MRS > 0**				
Coefficient	2.442	2.214	-0.144	-0.228
p-value	0.323	0.344	0.856	0.768
CI	[-2.450 7.333]	[-2.415 6.843]	[-1.718 1.431]	[-1.760 1.305]
**AOM > 2**				
Coefficient	**5.181**	**4.954**	1.385	1.302
p-value	**0.049**	**0.047**	0.102	0.113
CI	**[0.019 10.342]**	**[0.063 9.846]**	[-0.280 3.050]	[-0.314 2.919]
**LS_9_6**				
Coefficient	1.042	1.026	1.275	1.198
p-value	0.696	0.685	0.132	0.145
CI	[-4.254 6.337]	[-3.986 6.038]	[-0.394 2.945]	[-0.423 2.819]
**ELF_9_8**				
Coefficient	1.042	0.895	1.228	1.093
p-value	0.696	0.720	0.147	0.184
CI	[-4.254 6.337]	[-4.062 5.852]	[-0.443 2.900]	[-0.531 2.717]
**RSIBD**				
Coefficient	3.688	3.437	1.089	0.972
p-value	0.131	0.136	0.164	0.202
CI	[-1.128 8.505]	[-1.108 7.983]	[-0.455 2.634]	[-0.534 2.478]
**IBD presence**				
Coefficient	-2.228	-2.110	-1.198	-1.098
p-value	0.438	0.437	0.191	0.216
CI	[-7.928 3.472]	[-7.490 3.271]	[-3.007 0.612]	[-2.849 0.653]
**Anali**				
Coefficient	0.736	0.546	0.588	0.460
p-value	0.774	0.820	0.473	0.561
CI	[-4.353 5.826]	[-4.205 5.297]	[-1.035 2.211]	[-1.107 2.026]
**Cirrhosis**				

	PSC-PRO symptoms		PSC-PRO Total impact of symptoms	
	Available data	MI	Available data	MI
Coefficient	1.624	1.421	0.441	0.329
p-value	0.553	0.579	0.614	0.696
CI	[-3.807 7.055]	[-3.651 6.493]	[-1.297 2.180]	[-1.341 2.000]
**cT1 Whole Median**				
Coefficient	0.660	0.271	0.399	0.049
p-value	0.876	0.942	0.768	0.968
CI	[-7.742 9.061]	[-7.139 7.680]	[-2.288 3.085]	[-2.429 2.527]
Number of observations	75	80	75	80

b). Cross-sectional data analysis (n=78)

Year 1 PROMs by different risk groups (Multiple imputation)

	SF6D QoL		SF36 physical component summary		SF36 mental component summary	
	Available data	MI	Available data	MI	Available data	MI
**Extrahepatic disease**						
Coefficient	-0.085	-0.064	**-5.750**	-3.411	-2.929	-2.031
p-value	0.053	0.104	**0.038**	0.178	0.395	0.498
CI	[-0.171 0.001]	[-0.141 0.013]	**[-11.163 -0.337]**	[-8.426 1.604]	[-9.794 3.936]	[-7.995 3.933]
**Dominant stricture**						
Coefficient	**-0.101**	-0.058	**-8.394**	-5.325	-4.096	-2.155
p-value	**0.029**	0.170	**0.003**	0.051	0.258	0.499
CI	**[-0.191 -0.011]**	[-0.142 0.026]	**[-13.848 -2.939]**	[-10.674 0.025]	[-11.294 3.102]	[-8.492 4.182]
**ULNALP 1.5 risk**						
Coefficient	-0.027	-0.029	-2.271	-0.811	-1.562	-0.949
p-value	0.594	0.553	0.480	0.786	0.692	0.778
CI	[-0.129 0.075]	[-0.126 0.069]	[-8.688 4.147]	[-6.784 5.163]	[-9.427 6.304]	[-7.676 5.779]
**ULNALP 2.2 risk**						
Coefficient	0.008	-0.021	0.508	0.982	2.623	0.412
p-value	0.907	0.724	0.907	0.807	0.621	0.931
CI	[-0.130 0.146]	[-0.140 0.098]	[-8.199 9.215]	[-7.086 9.050]	[-7.984 13.231]	[-9.196 10.021]
**MRS > 0**						
Coefficient	-0.015	-0.027	-2.479	-1.616	-0.191	0.179
p-value	0.745	0.495	0.379	0.534	0.956	0.955
CI	[-0.104 0.075]	[-0.107 0.052]	[-8.092 3.135]	[-6.802 3.569]	[-7.103 6.721]	[-6.156 6.514]
**AOM > 2**						
Coefficient	-0.022	-0.042	-3.669	-2.457	0.058	-0.279

	SF6D QoL		SF36 physical component summary		SF36 mental component summary	
	Available data	MI	Available data	MI	Available data	MI
p-value	0.650	0.370	0.231	0.434	0.988	0.943
CI	[-0.120 0.075]	[-0.137 0.052]	[-9.751 2.414]	[-8.731 3.816]	[-7.483 7.600]	[-8.108 7.549]
**LS_9_6**						
Coefficient	-0.092	-0.067	**-6.536**	-3.465	**-7.800**	-4.203
p-value	0.054	0.135	**0.030**	0.220	**0.034**	0.219
CI	[-0.186 0.002]	[-0.156 0.022]	**[-12.412 - 0.659]**	[-9.063 2.132]	**[-14.994 - 0.607]**	[-10.984 2.578]
**ELF_9_8**						
Coefficient	-0.060	-0.057	-5.004	-2.910	-5.037	-2.740
p-value	0.219	0.215	0.100	0.334	0.177	0.476
CI	[-0.156 0.037]	[-0.148 0.034]	[-11.006 0.998]	[-8.912 3.092]	[-12.435 2.362]	[-10.447 4.966]
**RSIBD**						
Coefficient	-0.068	-0.044	-4.908	-2.625	-0.677	0.104
p-value	0.126	0.243	0.078	0.287	0.845	0.972
CI	[-0.155 0.020]	[-0.119 0.031]	[-10.385 0.570]	[-7.516 2.267]	[-7.586 6.232]	[-5.755 5.962]
**IBD presence**						
Coefficient	0.002	0.011	1.553	1.884	1.491	2.138
p-value	0.973	0.804	0.603	0.512	0.682	0.529
CI	[-0.093 0.096]	[-0.077 0.099]	[-4.404 7.510]	[-3.826 7.594]	[-5.792 8.774]	[-4.620 8.897]
**Anali**						
Coefficient	-0.034	-0.017	-4.112	-0.822	-2.519	0.273
p-value	0.475	0.709	0.164	0.776	0.489	0.935
CI	[-0.128 0.060]	[-0.108 0.074]	[-9.965 1.742]	[-6.616 4.971]	[-9.778 4.740]	[-6.377 6.923]
**Cirrhosis**						
Coefficient	-0.017	-0.014	-3.217	-1.462	-1.074	0.228
p-value	0.726	0.762	0.286	0.618	0.772	0.947
CI	[-0.113 0.079]	[-0.105 0.078]	[-9.211 2.777]	[-7.305 4.381]	[-8.476 6.328]	[-6.594 7.050]
**cT1 Whole Median**						
Coefficient	**-0.190**	**-0.154**	**-15.489**	**-12.677**	-7.209	-5.559
p-value	**0.004**	**0.013**	**0.000**	**0.002**	0.171	0.251
CI	**[-0.316 - 0.063]**	**[-0.274 - 0.034]**	**[-22.948 - 8.031]**	**[-20.403 - 4.950]**	[-17.636 3.218]	[-15.162 4.045]
Number of observations	50.000	78.000	50.000	78.000	50.000	78.000

	PSC-PRO symptoms		PSC-PRO Total impact of symptoms	
	Available data	MI	Available data	MI
**Extrahepatic disease**				

	PSC-PRO symptoms		PSC-PRO Total impact of symptoms	
	Available data	MI	Available data	MI
Coefficient	**5.269**	3.390	1.741	0.853
p-value	**0.016**	0.117	0.117	0.389
CI	**[1.046 9.493]**	[-0.882 7.662]	[-0.450 3.932]	[-1.114 2.820]
**Dominant stricture**				
Coefficient	**4.583**	2.698	**2.850**	1.980
p-value	**0.048**	0.245	**0.013**	0.070
CI	**[0.036 9.130]**	[-1.903 7.300]	**[0.628 5.072]**	[-0.167 4.128]
**ULNALP 1.5 risk**				
Coefficient	4.821	4.118	0.796	0.522
p-value	0.055	0.096	0.533	0.662
CI	[-0.100 9.742]	[-0.759 8.996]	[-1.755 3.346]	[-1.869 2.912]
**ULNALP 2.2 risk**				
Coefficient	**6.735**	5.122	0.258	0.184
p-value	**0.046**	0.133	0.882	0.907
CI	**[0.112 13.358]**	[-1.625 11.870]	[-3.198 3.713]	[-2.989 3.358]
**MRS > 0**				
Coefficient	1.080	1.520	1.197	1.123
p-value	0.630	0.490	0.284	0.270
CI	[-3.397 5.557]	[-2.865 5.905]	[-1.023 3.416]	[-0.897 3.144]
**AOM > 2**				
Coefficient	3.895	3.509	1.574	1.938
p-value	0.107	0.189	0.195	0.086
CI	[-0.869 8.660]	[-1.798 8.815]	[-0.834 3.982]	[-0.283 4.159]
**LS_9_6**				
Coefficient	3.895	3.493	**2.915**	**2.702**
p-value	0.107	0.176	**0.014**	**0.015**
CI	[-0.869 8.660]	[-1.626 8.613]	**[0.614 5.216]**	**[0.553 4.850]**
**ELF_9_8**				
Coefficient	3.895	3.368	**3.018**	**2.960**
p-value	0.107	0.191	**0.011**	**0.008**
CI	[-0.869 8.660]	[-1.746 8.482]	**[0.729 5.308]**	**[0.816 5.104]**
**RSIBD**				
Coefficient	3.640	2.751	1.223	0.863
p-value	0.100	0.208	0.273	0.369
CI	[-0.722 8.002]	[-1.576 7.077]	[-0.995 3.442]	[-1.043 2.768]
**IBD presence**				
Coefficient	4.152	3.697	-0.763	-0.575
p-value	0.075	0.113	0.519	0.603

	PSC-PRO symptoms		PSC-PRO Total impact of symptoms	
	Available data	MI	Available data	MI
CI	[-0.430 8.733]	[-0.895 8.290]	[-3.124 1.598]	[-2.769 1.620]
**Anali**				
Coefficient	1.374	1.533	1.825	1.834
p-value	0.561	0.513	0.119	0.085
CI	[-3.346 6.095]	[-3.141 6.206]	[-0.486 4.137]	[-0.263 3.931]
**Cirrhosis**				
Coefficient	-1.088	0.139	0.892	1.344
p-value	0.651	0.957	0.457	0.227
CI	[-5.889 3.712]	[-5.006 5.284]	[-1.502 3.287]	[-0.861 3.550]
**cT1 Whole Median**				
Coefficient	**11.659**	**9.174**	**3.682**	3.002
p-value	**0.000**	**0.010**	**0.029**	0.059
CI	**[5.639 17.679]**	**[2.322 16.027]**	**[0.395 6.970]**	[-0.114 6.117]
Number of observations	50.000	78.000	50.000	78.000
