# Supplementary material for: Association between non-invasive biomarkers and quality of life in Primary Sclerosing Cholangitis
Source: PLoS One. 2025 Nov 12;20(11):e0335642. doi: 10.1371/journal.pone.0335642 (PMC12611166; doi:10.1371/journal.pone.0335642)
Supplement: Attachment — Submitted filename: A rebuttal letter.docx [file pone.0335642.s017.docx]

Journal Requirements:

1. Please ensure that your manuscript meets PLOS ONE's style requirements, including those for file naming. The PLOS ONE style templates can be found at

<https://journals.plos.org/plosone/s/file?id=wjVg/PLOSOne_formatting_sample_main_body.pdf> and

[https://journals.plos.org/plosone/s/file?id=ba62/PLOSOne_formatting_sample_title_authors_affiliations.pdf](https://track.editorialmanager.com/CL0/https:%2F%2Fjournals.plos.org%2Fplosone%2Fs%2Ffile%3Fid=ba62%2FPLOSOne_formatting_sample_title_authors_affiliations.pdf/1/010f01983ca2bafd-273f76a8-0bb0-4e5a-a39a-04229cce8e8e-000000/9xenl7bYetgUq1yw09izTkZgywcrnwEpgjtcwLU-5ho=221)

AUTHORS’ REPLY: Thank you for the comment. We changed the manuscript to meet the style requirements, including changes of files name.

2. Thank you for stating the following financial disclosure:
“JD, JL, ELC, MP and AB supported by Oxford NIHR Biomedical Research Centre. (<https://oxfordbrc.nihr.ac.uk/>)

ELC receives funding from PSC Support and Oxford Health Service Research Committee.”

Please state what role the funders took in the study. If the funders had no role, please state: "The funders had no role in study design, data collection and analysis, decision to publish, or preparation of the manuscript."
If this statement is not correct you must amend it as needed.
Please include this amended Role of Funder statement in your cover letter; we will change the online submission form on your behalf.

AUTHORS’ REPLY: Thank you for the comment. We have now added the funders’ role into the Funding statement: "The funders had no role in study design, data collection and analysis, decision to publish, or preparation of the manuscript."

We also add one sentence in the cover letter: “We confirm that the funders had no role in study design, data collection and analysis, decision to publish, or preparation of the manuscript.”

3. Please expand the acronym “NIHR” (as indicated in your financial disclosure) so that it states the name of your funders in full.

This information should be included in your cover letter; we will change the online submission form on your behalf.

AUTHORS’ REPLY: Thank you for the comment. NIHR stands for National Institute for Health and Care Research which is now expanded in the paper. We have also added the funder’s information to the cover letter.

4. Thank you for stating the following in the Acknowledgments Section of your manuscript:
“This study is funded by Oxford NIHR BRC imaging theme.”

We note that you have provided funding information that is not currently declared in your Funding Statement. However, funding information should not appear in the Acknowledgments section or other areas of your manuscript. We will only publish funding information present in the Funding Statement section of the online submission form.
Please remove any funding-related text from the manuscript and let us know how you would like to update your Funding Statement. Currently, your Funding Statement reads as follows:
“JD, JL, ELC, MP and AB supported by Oxford NIHR Biomedical Research Centre. (<https://oxfordbrc.nihr.ac.uk/>)

ELC receives funding from PSC Support and Oxford Health Service Research Committee.”

Please include your amended statements within your cover letter; we will change the online submission form on your behalf.

AUTHORS’ REPLY: We have removed the funding information as requested from the manuscript (Funding statement and Conflict of interest disclosure) and added it to the cover letter.

5. Thank you for stating the following in the Competing Interests section:
“I have read the journal's policy and the authors of this manuscript have the following competing interests: MB is employed by Perspectum Ltd and is a shareholder in Perspectum Ltd, MP is a shareholder in Perspectum Ltd.

ELC: Speaking Fees: Advanz (Intercept), Albireo, Dr Falk Pharma, Gilead, GSK, Mirum; Consulting Fees: Advanz (Intercept), Amgen (Horizon Therapeutics), Ipsen, Mirum, Moderna, Sanofi, Zenus Pharma, Sail; Grant Support: Jansen, Innovate UK, PSC Support; Institutional Funding Support: BRC Oxford NIHR (UK), Oxford Health Service Research Committee Grant.”

We note that you received funding from a commercial source: Perspectum Ltd

Please provide an amended Competing Interests Statement that explicitly states this commercial funder, along with any other relevant declarations relating to employment, consultancy, patents, products in development, marketed products, etc.

Within this Competing Interests Statement, please confirm that this does not alter your adherence to all PLOS ONE policies on sharing data and materials by including the following statement: "This does not alter our adherence to PLOS ONE policies on sharing data and materials.” (as detailed online in our guide for authors <http://journals.plos.org/plosone/s/competing-interests>). If there are restrictions on sharing of data and/or materials, please state these. Please note that we cannot proceed with consideration of your article until this information has been declared.

Please include your amended Competing Interests Statement within your cover letter. We will change the online submission form on your behalf.

AUTHORS’ REPLY: Thank you for this comment and we apologise if this was not clear in the initially amended manuscript. We did not receive any funding from Perspectum. Perspectum provided analysis for MRCP+ and liver multiscan data as contribution in kind. We have added this to our conflict-of-interest statement in Cover letter.

6. Please include a separate caption for each figure in your manuscript.

AUTHORS’ REPLY: We have now included separate captions in the manuscript.

7. Please include captions for your Supporting Information files at the end of your manuscript, and update any in-text citations to match accordingly. Please see our Supporting Information guidelines for more information: <http://journals.plos.org/plosone/s/supporting-information>.

The captions of my supporting information are now at the end of my manuscript, and in the format the Journal asked.

8. Please review your reference list to ensure that it is complete and correct. If you have cited papers that have been retracted, please include the rationale for doing so in the manuscript text, or remove these references and replace them with relevant current references. Any changes to the reference list should be mentioned in the rebuttal letter that accompanies your revised manuscript. If you need to cite a retracted article, indicate the article’s retracted status in the References list and also include a citation and full reference for the retraction notice.

AUTHORS’ REPLY: We have reviewed the reference list.

[Note: HTML markup is below. Please do not edit.]

Reviewers' comments:

Reviewer's Responses to Questions

9. (09/09/2025) In this instance it seems there may be acceptable restrictions in place that prevent the public sharing of your minimal data. However, in line with our goal of ensuring long-term data availability to all interested researchers, PLOS’ Data Policy states that authors cannot be the sole named individuals responsible for ensuring data access (<http://journals.plos.org/plosone/s/data-availability#loc-acceptable-data-sharing-methods>).

AUTHORS’ REPLY: We thank the Editor for the helpful comments regarding data availability. The dataset used for all analyses is now publicly available on the Open Science Framework (OSF) (DOI: <https://doi.org/10.17605/OSF.IO/BH94N>). The Data Availability Statement and CL have been revised to reflect this update.

**Comments to the Author**

1. Is the manuscript technically sound, and do the data support the conclusions?

The manuscript must describe a technically sound piece of scientific research with data that supports the conclusions. Experiments must have been conducted rigorously, with appropriate controls, replication, and sample sizes. The conclusions must be drawn appropriately based on the data presented.

Reviewer #1: Yes

Reviewer #2: Yes

2. Has the statistical analysis been performed appropriately and rigorously?

Reviewer #1: Yes

Reviewer #2: Yes

3. Have the authors made all data underlying the findings in their manuscript fully available?

The [PLOS Data policy](http://track.editorialmanager.com/CL0/http:%2F%2Fwww.plosone.org%2Fstatic%2Fpolicies.action%23sharing/1/010f01983ca2bafd-273f76a8-0bb0-4e5a-a39a-04229cce8e8e-000000/XTerRirapJHx3XHXCofa5DCY141W4-HNknnoUwMBtro=221) requires authors to make all data underlying the findings described in their manuscript fully available without restriction, with rare exception (please refer to the Data Availability Statement in the manuscript PDF file). The data should be provided as part of the manuscript or its supporting information, or deposited to a public repository. For example, in addition to summary statistics, the data points behind means, medians and variance measures should be available. If there are restrictions on publicly sharing data—e.g. participant privacy or use of data from a third party—those must be specified.

Reviewer #1: Yes

Reviewer #2: Yes

4. Is the manuscript presented in an intelligible fashion and written in standard English?

PLOS ONE does not copyedit accepted manuscripts, so the language in submitted articles must be clear, correct, and unambiguous. Any typographical or grammatical errors should be corrected at revision, so please note any specific errors here.

Reviewer #1: Yes

Reviewer #2: Yes

5. Review Comments to the Author

Please use the space provided to explain your answers to the questions above. You may also include additional comments for the author, including concerns about dual publication, research ethics, or publication ethics. (Please upload your review as an attachment if it exceeds 20,000 characters)

Reviewer #1: Your study was well done and will be of interest to a spectrum of care givers, basic scientists, epidemiologists, pharmaceutical companies and governmental agencies. I have nothing further to add. .

Reviewer #2: The authors conducted a study with 80 PSC patients total and examined whether sveral biomarkers, MRCP+ metrics and risk scores correlate with changes in QoL. To my knowdledge there is no other study which analyzed this combination of markers. The authors collected data at basline and after 1 year. They have identified markers which are connected to a more advanced disease and the identified that an increase in liver stiffness correlates with reduced QoL indicators.

The limitations of this study are the sample size and the fact that they only received data at 2 timepoints and only with a one year gap.Since PSC is a slow progressing disease, one year is a short time. But, in the discussion the authors adressed the same concerns.

Otherwise the technical aspects and stastics are sound and reasonable, although the authors could eloborate more why they chose exactly those regression models. Moreover, they used standard QoL measurements and biomarkers/risk scores which are well documented. Whats new is the combinations of markers and the correlation to QoL.

From my perspective, a more eloborate statement regarding the impact of the study on clincal practice is missing. Why and how is it useful to now which markers are connected to a reduced QoL? Additionally, an outlook would be interesting, so whether the authors plan to repeat the study with more patients at longer intervals. Since they indicated that sampling is ongoing.

AUTHORS’ REPLY: We thank the reviewer for this important comment. We have revised our closing paragraph to emphasise the potential clinical implications of our work. We already provide suggestions for future work and we will update our results once our study is completed. At this point in time, we have not secured funding for a study extension beyond the 1-year follow up.

Our last paragraph (Page 22) now reads:

“In summary, PSC is a rare chronic liver disease that can affect quality of life and our study has potential important clinical implications. Knowing whether biomarkers are associated to disease severity as well as QoL can inform clinicians and health system how to use biomarkers in clinical care. By choosing to use tools that can inform both for the disease severity and the impact this is having on the quality of life care can become more efficient. For example, by employing a tool that can measure bith disease severity and QoL can negate the need for using separate tools for monitoring disease severity and questionnaires to measure QoL. Surrogate biomarkers may be helpful in stratifying people with PSC according to their quality of life besides the risk of disease progression. We found several markers including RSIBD measured by MRCP+ and liver cT1 measured by Liver MultiScan to be cross sectionally associated with QoL. In addition, changes in LS were also associated with changes in QoL in people with PSC. Further work should evaluate how the wealth of quantitative MRI data relate to quality of life to further inform the potential role of these parameters in the management of people with PSC. PROMs are important beyond clinical and imaging findings and provide key insight into people with PSC’ perspectives and lived experiences.”

Points to revise:
- Table 1: ¶ this symbol was used twice for different markers, which is a bit confusing

AUTHORS’ REPLY: Thank you for the comment, in Table 1, ¶ symbol is now only used for ULN.

- 2.3.1: Module 1 consists of 13 question with 10 being the highest score. Should it not add up to 130 and not 120?

AUTHORS’ REPLY: The question: “Are you currently experiencing a flare-up of your PSC symptoms (also known as acute cholangitis or an infection in the bile ducts)? Y/N”, does not produce a score.

- 2.3.2: the information is in the manual, but shortly mention which scales were combined for easier understanding

AUTHORS’ REPLY: We have added the following sentence (page 9):

“Physical component summary (PCS) and mental component summary (MCS) scores are aggregated by applying a scoring algorithm combines weighted contributions from all eight health scales.”

- 3.1: while the IBD prescence is not significant between the baseline and 1 year, authors should mention that 10 % is still a modest difference and might be significant when a larger sample size is used

AUTHORS’ REPLY: We agree with the reviewer and have added the following to our discussion (page 22):

“Furthermore, there was a numerical difference of 10% in the proportion of participants with IBD at baseline and 1 year. While this did not reach statistical significance due to small numbers in our study, it may become significant when larger studies are considered.”

6. PLOS authors have the option to publish the peer review history of their article ([what does this mean?](https://track.editorialmanager.com/CL0/https:%2F%2Fjournals.plos.org%2Fplosone%2Fs%2Feditorial-and-peer-review-process%23loc-peer-review-history/1/010f01983ca2bafd-273f76a8-0bb0-4e5a-a39a-04229cce8e8e-000000/kkzyTz_xJQqZ0X8CclJFxVeAp-kA4yc6cwD6zwoqhR8=221)). If published, this will include your full peer review and any attached files.


If you choose “no”, your identity will remain anonymous but your review may still be made public.


**Do you want your identity to be public for this peer review?** For information about this choice, including consent withdrawal, please see our [Privacy Policy](https://track.editorialmanager.com/CL0/https:%2F%2Fwww.plos.org%2Fprivacy-policy/1/010f01983ca2bafd-273f76a8-0bb0-4e5a-a39a-04229cce8e8e-000000/wWOMv84xDNDQdGbfjzaLHh6gniQkdIqwkZkaNEIloZc=221).

Reviewer #1: No

Reviewer #2: No
